# Revolutionizing Precision Medicine: Exploring Wearable Sensors for Therapeutic Drug Monitoring and Personalized Therapy

**DOI:** 10.3390/bios13070726

**Published:** 2023-07-12

**Authors:** Yuqiao Liu, Junmin Li, Shenghao Xiao, Yanhui Liu, Mingxia Bai, Lixiu Gong, Jiaqian Zhao, Dajing Chen

**Affiliations:** 1 School of Pharmacy, Hangzhou Normal University, Hangzhou 311121, China; 2021112012291@stu.hznu.edu.cn (Y.L.); 2021112012302@stu.hznu.edu.cn (J.L.); 2022112025100@stu.hznu.edu.cn (S.X.); 2022112025018@stu.hznu.edu.cn (Y.L.); 2022112025030@stu.hznu.edu.cn (M.B.); 2021011012002@stu.hznu.edu.cn (L.G.); 2 College of Biomedical Engineering & Instrument Science, Zhejiang University, Hangzhou 310007, China

**Keywords:** wearable sensors, drug analysis, personalized medicine, therapeutic drug monitoring

## Abstract

Precision medicine, particularly therapeutic drug monitoring (TDM), is essential for optimizing drug dosage and minimizing toxicity. However, current TDM methods have limitations, including the need for skilled operators, patient discomfort, and the inability to monitor dynamic drug level changes. In recent years, wearable sensors have emerged as a promising solution for drug monitoring. These sensors offer real-time and continuous measurement of drug concentrations in biofluids, enabling personalized medicine and reducing the risk of toxicity. This review provides an overview of drugs detectable by wearable sensors and explores biosensing technologies that can enable drug monitoring in the future. It presents a comparative analysis of multiple biosensing technologies and evaluates their strengths and limitations for integration into wearable detection systems. The promising capabilities of wearable sensors for real-time and continuous drug monitoring offer revolutionary advancements in diagnostic tools, supporting personalized medicine and optimal therapeutic effects. Wearable sensors are poised to become essential components of healthcare systems, catering to the diverse needs of patients and reducing healthcare costs.

## 1. Introduction

Throughout the ongoing battle against illnesses, humans have gradually accumulated substantial experience in drug usage, resulting in a constant enrichment of our pharmacological practice. The use of drugs has become deeply ingrained in our society, assuming a pivotal role in disease treatment and physical regulation [1,2,3,4]. However, due to the physiologically-based pharmacokinetics, it is imperative for each p-atient to determine the appropriate dosage based on factors such as absorption, distribution, metabolism, and elimination rates in order to achieve optimal therapeutic effects [5]. Therefore, the development of precision medicine holds immense significance in mitigating drug toxicity.

Therapeutic drug monitoring (TDM) serves as a valuable approach within precision medicine, effectively minimizing drug side effects arising from individual differences. TDM involves the modern analytical techniques guided by pharmacokinetic (PK) principles to quantitatively determine drug and metabolite concentrations in patients’ biofluids post-treatment. TDM enables the design or adjustment of personalized drug delivery plans, thereby enhancing treatment efficacy, minimizing drug side effects, and facilitating personalized medicine [6,7]. To date, various techniques such as chromatography [8,9], immunoassay [10], nuclear magnetic resonance [11], isotope tracing [12], and capillary electrophoresis [13] have been employed to measure drug concentrations in biofluids for TDM purposes. TDM also aims to facilitate the development of precision drugs and personalized medicine with its high accuracy and low detection limits [14].

However, several limitations associated with TDM warrant attention. Firstly, TDM requires skilled operators to maintain complex instruments, and frequent blood sampling may cause discomfort for patients. Additionally, the specific storage requirements for biofluid samples could compromise the detecting accuracy by inducing drug degradation and transformation within the samples [15]. Furthermore, most TDM methods capture drug concentrations at a specific time, lacking the ability to monitor dynamic changes in drug levels continuously. Consequently, developing novel techniques for real-time in vivo monitoring of therapeutic drugs becomes imperative to overcome these limitations and enhance drug monitoring efficiency [16].

In recent years, the potential of wearable sensors for biomedical applications and health monitoring has drawn increasing attention [17]. These sensors have achieved significant advancements in miniaturization, multifunction, and algorithm, owing to the development of integrated devices and artificial intelligence. By enabling non-invasive or minimally invasive sample collection, wearable sensors possess the capability to monitor physiological signals, facilitate early disease diagnosis, and enable remote monitoring of various conditions. Furthermore, wearable sensors can play a crucial role in drug concentration monitoring within the blood and other biofluids, providing real-time signal transmission to assist patients in regulating drug dosage and minimizing the risk of drug toxicity. Additionally, these sensors can continuously monitor dynamic changes in drug levels over extended periods, supplying vital data necessary for optimal therapeutic effects. As a result, wearable multifunctional sensors are poised to become an essential component of healthcare systems, effectively catering to the personalized medicine requirements of diverse patients while reducing resource waste and associated healthcare costs.

**Figure 1:**
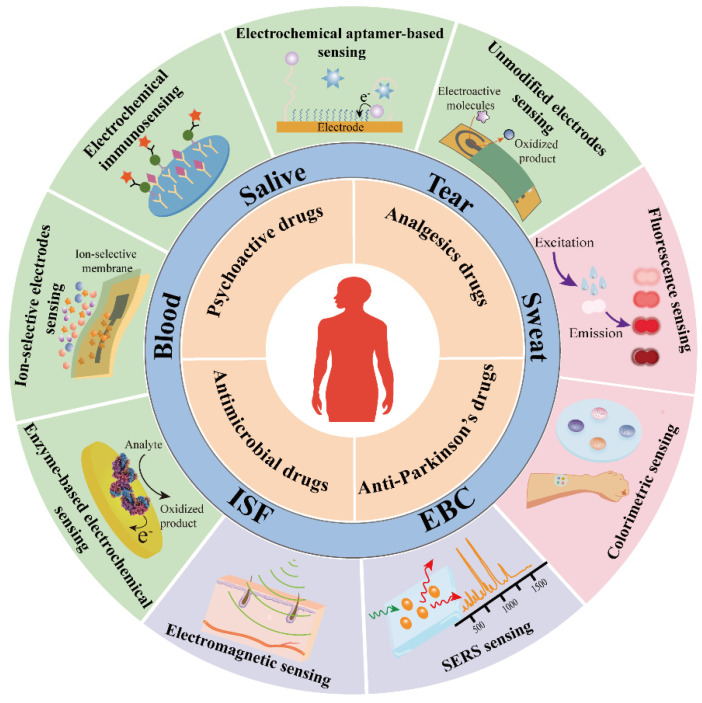
The schematic illustration depicting the four groups of drugs that can currently be wearable for monitoring, some biofluids for monitoring, and various available wearable monitoring sensing technologies.

This review offered an overview of wearable sensors for drug detection and explored emerging biosensing technologies that hold potential for future drug monitoring applications, including disease prevention, early diagnostic, therapeutic drug monitoring, auxiliary treatment, evaluation of treatment effects, and long-term management of chronic diseases [18,19] (Figure 1). The second section presented a concise summary of the four primary drug categories currently detectable by wearable sensors, emphasizing their clinical significance. When employed in drug monitoring applications, we conducted a comparative analysis of multiple biosensing technologies, considering their linear concentration range, operating range, interference resistance, and detection lifespan. In the third section, we provided a comprehensive overview of current biosensing technologies, evaluating their strengths and limitations as well as their potential to be integrated into wearable detection systems. Through this review, we aimed to highlight the most promising wearable drug monitoring technologies. In therapeutic drug monitoring, the real-time and continuous measurement capabilities offered by these technologies will undoubtedly catalyze the revolutionary advancement of diagnostic tools and provide robust support for personalized medicine.

## 2. Wearable Monitoring of Drugs

Therapeutic drug monitoring is an increasingly vital area with significant potential for enhancing patient outcomes. Nevertheless, several challenges, such as non-linear pharmacokinetics, low therapeutic indices, narrow safety ranges, and the potential for life-threatening side effects, have limited the successful implementation of wearable monitoring systems for drug monitoring. Conventional analytical methods for measuring in vivo drug concentrations require precise timing of blood sampling to accurately determine steady-state concentrations. Table 1 summarizes therapeutic and toxic concentrations for a number of representative drugs. In contrast, wearable technology has the capacity to revolutionize drug therapy by enabling real-time monitoring of drug concentration changes, thereby enhancing detection accuracy. In this section, we present a comprehensive overview of sensing technologies for drug molecules and the corresponding wearable sensors developed for monitoring anti-Parkinson’s drugs, antibiotics, analgesics, and neuroleptics based on the current research literature.

### 2.1. Anti-Parkinson’s Drugs

Parkinson’s disease (PD) is a neurodegenerative disorder primarily affecting middle-aged and elderly individuals, with a prevalence second only to Alzheimer’s disease [44]. PD patients have fewer nigrostriatal dopaminergic neurons in their brains, leading to motor symptoms, including resting tremor, bradykinesia, rigidity, postural instability, and impaired self-care [45,46,47]. The current gold standard for improving early disease symptoms is levodopa (L-Dopa), which serves as a dopamine precursor but lacks inherent pharmacological activity. After being catalyzed by dopamine decarboxylase, L-Dopa is converted to dopamine, a vital neurotransmitter that enhances nociceptor function and regulates motor neuron pathways [48]. Despite its effectiveness in early-stage PD treatment, L-Dopa’s pharmacokinetics can be significantly influenced by factors such as dietary intake, age, gender, and prior dosing history [49].

L-Dopa overdose may cause depression by elevating malondialdehyde levels. Therefore, wearable sensors for continuous L-Dopa monitoring have potential clinical applications by enabling accurate identification of individual drug metabolism differences and dosage adjustments [50]. In wearable sensors, sweat-based detection of L-Dopa is a promising approach. Researchers have used high-performance liquid chromatography to simultaneously measure blood drug concentrations, which validates the accuracy of the sweat detection process. One study reported a correlation of 0.678 between sweat and blood L-Dopa concentrations [51]. Dopamine in sweat can be detected by electrochemical methods, including enzyme-based chronoamperometry methods or cyclic voltammetry based on inorganic materials.

**Figure 2:**
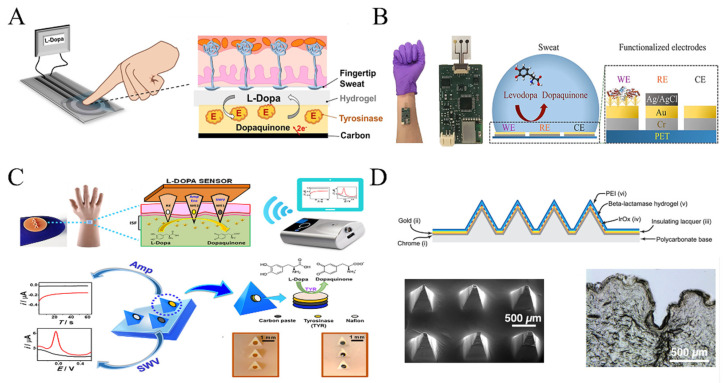
Wearable sensor for detecting L-Dopa and β-Lactam antimicrobial drugs: (**A**,**B**) Wearable electrochemical sensor based on tyrosinase catalysis to monitor L-Dopa in sweat (adapted from [52,55]); (**C**) Wearable electrochemical sensor based on voltammetry-based sensing modalities to monitor L-Dopa in interstitial fluid (adapted from [56]); (**D**) Wearable microneedle sensor based on β-Lactam enzyme catalysis to monitor β-Lactam antimicrobial drugs in interstitial fluid (adapted from [57]).

Moon et al. developed a wearable enzyme-based electrochemical biosensor for the real-time detection of L-Dopa in sweat [52]. The sensor utilized a screen-printed carbon paste substrate and immobilized tyrosinase on the surface to create a specific working electrode. By collecting sweat with a hydrogel covering the finger, the electrode detected L-Dopa through oxidation by tyrosinase, generating an electrochemical signal (Figure 2A). The sensors showed a minimum detection limit of 300 nM L-Dopa and exhibited similar pharmacokinetic profiles to blood samples. The wearable sensor also exhibited high selectivity for L-Dopa, with a signal response to C-Dopa that is only 5% of that of L-Dopa. This non-invasive approach holds potential for monitoring drug pharmacokinetics and can be extended to other important drugs.

The real-time detection of L-Dopa through wearable enzyme-based sensors has shown promising promising results. However, long-term enzyme stability has remained a significant concern for researchers [53,54]. To address this challenge, Xiao et al. reported a noninvasive and wearable enzyme-based electrochemical sensor for detecting L-Dopa in sweat based on metal-organic frameworks (MOFs) [55]. Zeolite imidazolate framework (ZIF-8) and tyrosinase were co-precipitated on the surface of graphene oxide (GO), resulting in ZIF-8/GO composites with a wide linear response range of 1 to 95 µM and a lower detection limit of 0.45 µM, indicating high sensitivity and stability (Figure 2B).

A novel sensing paradigm has emerged in the pursuit of achieving accurate signal measurements of L-Dopa. Goud et al. reported a minimally invasive microneedle sensing platform for orthogonal electrochemical monitoring of L-Dopa [56]. This platform utilized two sensing modes, redox and enzyme-catalyzed, simultaneously on both unmodified and tyrosinase-modified carbon paste microneedle electrodes. These parallel and independent modes enabled non-enzymatic voltammetry and enzyme-catalyzed amperometric detection of L-Dopa, resulting in an impressive L-Dopa detection limit of approximately 0.5 μM (Figure 2C).

In summary, wearable detection of levodopa primarily relies on the use of specific enzymes to enhance electrode functionality, thereby improving selectivity and sensitivity. However, the practical application of these methods is limited by the inherent instability of enzymes. As a result, current detection methods have primarily emphasized the sensitivity of individual measurements, while long-term sensor sensitivity remains an area that requires further exploration.

### 2.2. Antimicrobial Drugs

Antibiotics play a vital role in treating infectious diseases, such as sepsis, burns, organ transplants, and obesity, by interfering with the growth and development of bacteria. In the early stages of the disease, antibiotics exhibit promising therapeutic effects, and timely administration can save patients’ lives. However, in the clinical use of antibiotics, the pharmacokinetics of individual patients greatly varied, making it difficult to assess the appropriate dose. Although several biosensing technologies are available for antibiotic detection in vitro, wearable detection has only been implemented for vancomycin, kanamycin, tobramycin, and phenoxymethylpenicillin, utilizing interstitial fluid and blood as test samples, where the drug concentration quickly equilibrates to reflect blood drug concentration levels (Figure 2D).

Vancomycin is a crucial antibiotic used to treat infections caused by penicillin-resistant staphylococci and can serve as an alternative medication for patients with severe β-lactam antibiotic allergy [58]. However, it has a very narrow therapeutic window (5–40 μg mL^−1^) and if inappropriately dosed for a period, it may result in adverse reactions such as ototoxicity, nephrotoxicity, peripheral venous complications, and allergies [59]. Due to its non-linear pharmacokinetics, indirect measurement of vancomycin’s peak and trough concentrations may have limited practicality. The current wearable detection technologies for vancomycin have primarily focused on electrochemical, aptamer-based (E-AB) sensors. In a recent study by Dauphin-Ducharme et al. [30], an E-AB sensor was placed in the vein of a rat through a catheter to enable real-time detection of vancomycin concentration in plasma. Aptamers were immobilized onto the working electrode surface, with methylene blue serving as the redox reporter. Upon binding of vancomycin to the aptamers, the distance between the methylene blue and the electrode increases, or the transfer of electrolyte is hindered, resulting in a decrease in the electrochemical signal. By analyzing the change in electron transfer rate, the concentration of vancomycin can be derived. The sensor exhibited a stable response signal 9 s after injection of 10 μM vancomycin, with a linear detection range of approximately 6–35 μM. These results successfully demonstrated the high signal accuracy of the E-AB sensor. Moreover, under controlled administration, the E-AB sensor maintained a therapeutic window concentration of ±2 μM for several hours (<6 h), significantly improving the therapeutic effect.

**Figure 3:**
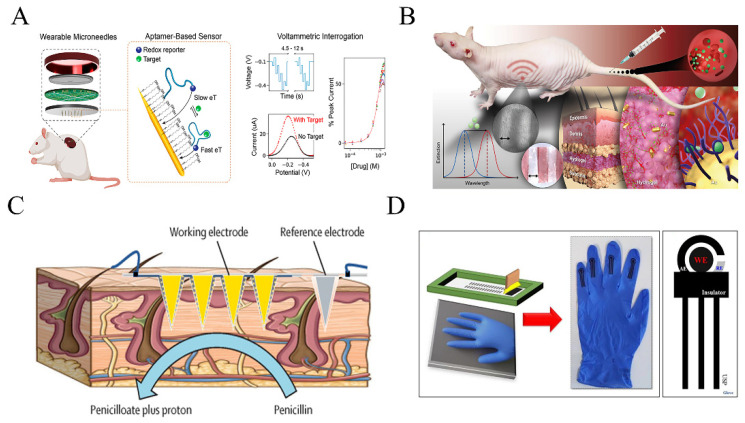
Wearable sensors for detecting antimicrobial drugs and psychoactive drugs: (**A**) Wearable electrochemical aptamer-based sensors to monitor tobramycin in interstitial fluid (adapted from [61]); (**B**) A novel gold nanoparticle-based implanted sensor for kanamycin concentration monitoring (adapted from [62]); (**C**) A wearable microneedle sensor based on β-Lactam enzyme catalysis to monitor phenoxymethylpenicillin in interstitial fluid (adapted from [65]); (**D**) Wearable glove sensors to monitor psychoactive drugs in sweat (adapted from [66]).

Tobramycin is a commonly used drug to treat cystic fibrosis (CF) caused by *Pseudomonas aeruginosa.* Its therapeutic window is relatively narrow, typically requiring concentrations of 4–6 μg mL^−1^ in the blood [34]. Unfortunately, a common side effect is hearing loss, which can fluctuate [60]. To address this challenge, researchers have developed an electrochemical aptamer-based detection technology using microneedles. 3D printing was utilized to fabricate poly(methyl methacrylate) microneedle arrays, which were coated with conductive layers and sensitive elements. Tobramycin aptamers were then bonded to the microneedle electrodes to enable the detection of tobramycin in interstitial fluids. The elimination half-life of tobramycin was 23 ± 2 min, consistent with the results measured in blood [61]. This approach offers a potential solution to monitor the tobramycin concentration in real-time, enabling more precise and effective treatments for CF patients (Figure 3A).

Kanamycin, an aminoglycoside antibiotic isolated from *Streptomyces kanamyceticus*, is mainly known for interfering with ribosomal RNA and the inhibiting of bacterial protein synthesis. It also destroys the integrity of bacterial cell membranes and has been proven effective against infections caused by Gram-negative bacteria. However, the effective concentration range of kanamycin is narrow, ranging from 15–30 μg mL^−1^. As kanamycin cannot be metabolized in the body and is mainly excreted through glomerular filtration, overdose can lead to severe renal toxicity, neuromuscular blockade, and allergic reactions. Unfortunately, there is no specific antagonist to treat kanamycin overdose, and the only way to remove it from the body is through large amounts of water supplementation, followed by hemodialysis or peritoneal dialysis. To address this issue, wearable detection methods of kanamycin have been developed, including electrochemical aptamer detection and photoacoustic imaging. Chien et al. fabricated a chronoamperometry sensor implantable in a vein to directly measure changes in electron transfer kinetics at the far end of the aptamer [59]. During the measurement process, a sample-and-hold circuit was employed to decrease the device power consumption from 5.2 mW to 0.22 mW and improve the molecular detection limitation from 57 to 12.3 μM. Kaefer et al. developed an optical imaging method to detect kanamycin concentrations in real time [62]. Gold nanoparticles were embedded into macro-porous hydrogel scaffolds and exhibited excellent biocompatibility. The hydrogels facilitated the growth of cells and blood vessels within their structure, overcoming the obstructing physical exchange between the sensor and adjacent tissues. By leveraging the plasmon effect, the gold nanoparticles absorbed and scattered near-infrared light of specific wavelengths and utilized the plasmon effect to detect a variety of drug molecules. Specifically, the concentration of kanamycin was determined by inducing a change in the refractive index of the gold nanoparticles, resulting in a shift in the plasma absorption wavelength. This implantable sensor demonstrated long-term stability and enabled continuous monitoring of the pharmacokinetic process of kanamycin in vivo for several weeks (Figure 3B).

Phenoxymethylpenicillin is a semi-synthetic penicillin with a similar antimicrobial spectrum to penicillin that is effective against Gram-positive bacteria. Recently, microneedle electrochemical sensors based on β-lactamases have been developed for the detection of phenoxymethylpenicillin [63]. The polycarbonate microneedle surface was plated with gold to enhance its conductivity, followed by iridium oxide coating as a pH-sensitive layer, and a hydrogel layer containing β-lactamase was applied to the microneedle array. When the sensor was inserted into the skin, phenoxymethylpenicillin in interstitial fluid diffused through the hydrogel and was hydrolyzed by β-lactamase to penicillin thiazoles and protons. This reaction caused a decrease in the local pH of the sensor, which disrupted the oxidation equilibrium of iridium oxide and induced a change in the current (Figure 3C).

The importance of wearable detection of antibiotics in preserving human health cannot be overstated, particularly in light of the widespread use of antibiotics to treat infectious diseases and the growing prevalence of antibiotic-resistant bacteria [64]. While combination therapy involving multiple antibiotics can enhance therapeutic efficacy, it also carries the inherent risk of adverse reactions, potentially resulting in severe consequences. Consequently, the advancement of non-invasive sensing and wearable detection techniques holds great promise for optimizing drug dosages and mitigating the emergence of drug resistance.

### 2.3. Analgesic Drugs

Acetaminophen (APAP) is an analgesic and antipyretic drug prone to overdose during acute fever, resulting in hepatic centrilobular necrosis [67]. Upon entry into the body, APAP is metabolized by CYP-dependent cytochrome P450 to produce the highly cytotoxic n-acetyl-p-benzoquinone imine (NAPQI). NAPQI initially binds with glutathione in the liver, and after depletion of glutathione, it binds to the mitochondrial proteins, interfering with their normal function and causing irreversible liver damage [68,69]. Due to the absence of effective antidotes, preventing APAP overdose is the most efficient approach. Given APAP’s relatively short half-life, timely detection of blood drug peaks by blood sampling may be difficult, and APAP concentrations exceeding 1.1 μM may lead to hepatotoxicity [70]. Wearable APAP sensors have been developed, which employ differential pulse voltammetry (DPV) to measure the concentration of APAP in sweat and saliva, capitalizing on its electrochemical activity.

The presence of electrochemically active interferents in sweat can lead to the overlapping of signal peaks between target molecules, resulting in distorted signals that reduce the sensitivity of APAP detection. To address this challenge and effectively separate the redox peaks of interferents from APAP, Lin et al. used Nafion-coated and hydrogen-terminated boron-doped diamond electrodes (Nafion/H-BDDE) to construct the sensing interface [71]. Voltammetry was used to detect the redox peak of APAP, and the concentration change of the target molecule could be reflected by analyzing the peak strength. The Nafion/H-BDDE sensing interface utilized surface engineering strategies to reduce the adsorption of other electrochemical active molecules, effectively preventing the signal peaks of target molecules from being buried and generating distorted signals. The modified sensing interface could separate the target molecule and the redox peak of the interfering substance, accurately measured the electron transfer reaction rate constant and the concentration of APAP in sweat and saliva, and its detection limit can reach 1 μM. There was a similar dynamic distribution in the two matrices, indicating a similar distribution mechanism of analytes from the blood. The sensing mechanisms could also inspire researchers to monitor electrochemical active molecules and broaden the scope of drug monitoring.

A wearable sensor integrated onto plastic gloves has been developed for the detection of APAP in sweat and saliva. This sensor utilizes screen-printed carbonaceous nanomaterials to facilitate the electrooxidation of APAP [66]. Notably, the wearable glove sensor exhibits excellent stretching and bending capabilities, making it suitable for practical applications. The APAP detection limit reached 2.47 × 10^−7^ M (Figure 3D), which falls within the clinically relevant concentration range for therapeutic drugs. Furthermore, the glove sensor mitigates the risk of infection associated with prolonged wearing and is particularly well-suited for individuals with fragile and sensitive skin [70]. By simply sliding the glove sensor across the skin surface, real-time monitoring of drug molecule concentrations in biofluids can be achieved, minimizing the potential for sample contamination and molecule degradation [72].

**Figure 4:**
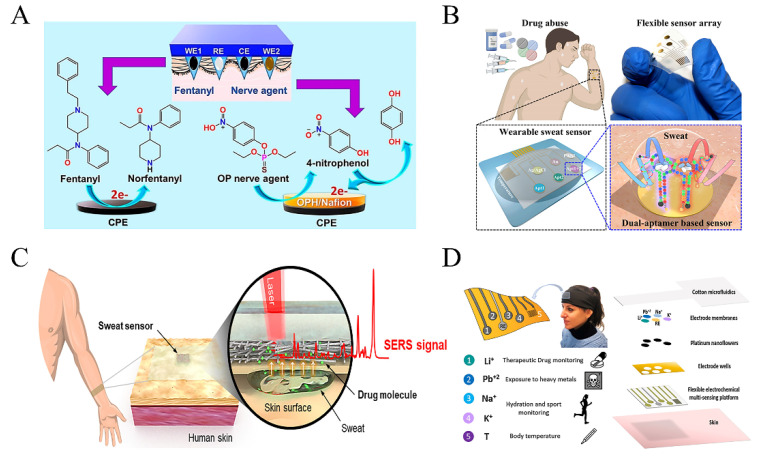
Wearable sensor for detecting antimicrobial drugs and psychoactive drugs: (**A**) A wearable microneedle sensor based on square wave voltammetry to monitor fentanyl in interstitial fluid (adapted from [65]); (**B**) Wearable electrochemical dual-aptamer-based sensor to monitor cathinone in sweat (adapted from [94]); (**C**) Wearable SERS patch sensor to monitor psychoactive drugs in sweat (adapted from [98]); (**D**) Wearable electrochemical sensor to monitor sweat ion concentration (adapted from [99]).

Fentanyl, a synthetic opioid used for anesthesia and analgesia, possesses pharmacological effects similar to morphine [73,74]. However, due to its narrow safe concentration range, it requires careful monitoring during its administration. The safe concentration range for analgesia is 1–3 mg L^−1^, and concentrations above 5 mg L^−1^ can easily lead to hypoxia, respiratory failure, and death [37,75,76]. Although fentanyl has a shorter onset time compared to other analgesic drugs, its lipophilicity increases the risk of exceeding the safe concentration range, leading to serious toxic reactions [77]. Mishra et al. developed a wearable microneedle electrochemical sensor that analyzes the intensity of redox peaks to detect fentanyl concentration [65]. Similar work was done by Joshi et al., who used 3D printed E-Shell 200 materials to fabricate a hollow microneedle platform for fentanyl detection [78]. The platform responded linearly with a dynamic detection range between 6.4 and 51.2 µg L^−1^ and an LOD value of 9.2 µg L^−1^ (Figure 4A).

The development of wearable sensors utilizing body fluids as detection samples has successfully generated pharmacokinetic curves for analgesic drugs in various bodily fluids, showcasing their real-time and dependable quantitative capabilities. This holds great promise for future medical monitoring applications, enabling timely intervention and prevention of adverse events such as liver failure, respiratory failure, and even fatal outcomes resulting from drug overdose.

### 2.4. Psychoactive Drugs

Abuse of psychoactive drugs has emerged as a major global concern because it poses a serious threat to public health, social stability, and economic growth [79,80,81]. Psychoactive drugs exert their effects by binding to specific receptors in the central nervous system, resulting in euphoria and excitement [82]. Therefore, timely monitoring of the blood concentration of psychoactive drugs is necessary to ensure the safety and effectiveness of treatment [83]. Existing testing technologies require collecting samples and subsequent in vitro testing, which is relatively cumbersome and time-consuming [84]. Recently, wearable sensors that use sweat as a sample have been developed to detect psychoactive drugs. These sensors employ electrochemical or surface-enhanced Raman spectroscopy sensing technologies to achieve continuous and quantitative monitoring of different drugs based on their unique chemical signatures.

Caffeine, the most widely consumed psychoactive substance in daily life, is considered relatively safe in daily intake. However, the chronic overdose of caffeine can lead to several health problems, including rhabdomyolysis and chronic kidney failure [85]. Toxicity can occur at caffeine concentrations exceeding 15 mg L^−1^ in the blood [86]. Therefore, researchers have studied wearable detection of caffeine. Researchers have thus explored the potential of wearable devices for monitoring caffeine levels. Tai et al. developed a flexible vinyl terephthalate substrate with a carbon nanotube-modified working electrode to detect caffeine levels in sweat [87]. Caffeine can be oxidized on a working electrode with a sensitivity of about 110 nA mm^−1^ at 1.4 V potential. During the measurement process, the authors observed that the peak concentration of caffeine appeared 60 min after oral intake, which is consistent with the previous literature reporting on caffeine metabolism [88,89,90,91].

Synthetic cathinone is a class of psychoactive substances that are obtained by modifying natural cathinone and includes about 30 different compounds. These drugs primarily promote sympathetic nerve stimulation and are commonly used for recreational purposes, leading to restlessness, aggressive behavior, and violent tendencies [92,93]. Zhang et al. reported on an E-AB sensor with two adapters (Apt1 and Apt2) capable of accurately identifying six different types of synthetic cathinone in sweat. The researchers prepared three working electrodes coated with Apt1, Apt2, and a mixture of both to verify the ability of each to detect multiple psychoactive drugs. Mixed aptamers carried more negative charges, resulting in diverse folded structures with higher sensitivity, recognition ability, and anti-interference ability. The two adapters showed high specificity and low cross-target reactivity, indicating their potential for the accurate detection of synthetic cathinone in sweat [94] (Figure 4B).

Methamphetamine is a potent central nervous system stimulant and is the primary component of methamphetamine. The abuse of methamphetamine is widespread, and doses exceeding 50 mg can cause neurotoxicity, acute and chronic cardiovascular complications [95,96,97]. In order to monitor and combat drug abuse, wearable sensors have been developed using 2-Fluoromethamphetamine (2-FMA) as a substitute for methamphetamine. Koh et al. utilized surface-enhanced Raman spectroscopy (SERS) to detect 2-FMA in sweat [98]. To improve detection accuracy, silk fibroin film (SFF) was employed to prepare sweat absorption pads, which not only possessed blocking properties but also facilitated the long-term retention of drug molecules in the patch. The incorporation of silver nanowires (AgNWs) into the silk fibroin film (SFF) further enhanced the intensity of the Raman signals. During the detection process, a portable Raman spectrometer was used to irradiate the patch, generating SERS signals that were subsequently processed and converted into the corresponding drug concentration. In addition, the researchers verified the drug concentrations using 2-FMA as a fluorescent probe, which yielded consistent results with the SERS patch (Figure 4C).

The diverse range of psychoactive drugs and the concurrent use of multiple substances by users to pursue excitement presents significant challenges for wearable drug detection. It necessitates the development of highly selective sensing technologies capable of detecting multiple drugs simultaneously. However, the majority of existing sensing technologies fall short in meeting these detection requirements. Therefore, it is crucial to optimize existing sensing technologies or develop new ones to broaden the scope of drug monitoring, especially for the monitoring of psychoactive ionic drugs (Figure 4D), which will greatly contribute to health and law enforcement monitoring work.

## 3. The Source of Biofluids and Continuous Sensing Technologies

In the past decade, wearable sensors have undergone rapid growth in the fields of health and drug monitoring, evolving intelligent sampling, analysis, and diagnosis [100]. By continuously monitoring changes in various physiological signals, wearable sensors can aid patients in taking more reasonable and effective measures to meet the demand for personalized treatment. There are three main categories of sensing technologies based on their location on the body: non-contact sensing, non-invasive contact sensing, and invasive sensing (which includes sensor tags in the blood circulatory system). In this chapter, we will discuss the technical characteristics, development status, and prospective development directions of wearable sensors. With the endeavors directed towards sensor advancements, personalized and precise medicine will soon become a reality.

### 3.1. Non-Contact Sensing Technology

Wearable non-contact sensing technology has gained attention as a promising approach for continuous drug monitoring without direct contact with biofluids. It can be divided into various types based on the physical parameters analyzed such as morphology, spectrum, and heat distribution. Non-contact sensing avoids the issues of sweat evaporation and contamination during collection and enables the detection of multiple drug molecules simultaneously. Consequently, it has a high potential for market acceptance and adoption by patients who take multiple drugs simultaneously. Researchers have developed various non-contact sensing methods, including optical and electromagnetic sensing, which provide results by measuring and analyzing changes in optical signals or electromagnetic fields when interacting with biological material. Compared with other detection technologies, non-contact sensing devices are more user-friendly and easily operated, rendering them an attractive option for drug monitoring in personalized medicine.

#### 3.1.1. Optical Sensing Technology

Non-contact optical sensing technology is a vital tool in drug monitoring due to its high sensitivity, accuracy, and resistance to electromagnetic interference. It has been widely used to monitor various physiological signals, such as respiration, heart rate, blood pressure, and blood glucose. In this technology, the incident light interacts with the target molecules, generating reflected or refracted light. The differences in intensity and wavelength between the refracted and incident light are used to identify characteristic absorption peaks. In wearable optical sensing technology, surface-enhanced Raman spectroscopy and infrared spectroscopy have been utilized to analyze the drug molecules in vivo, although most studies focus on body metabolites rather than drug monitoring.

**Figure 5:**
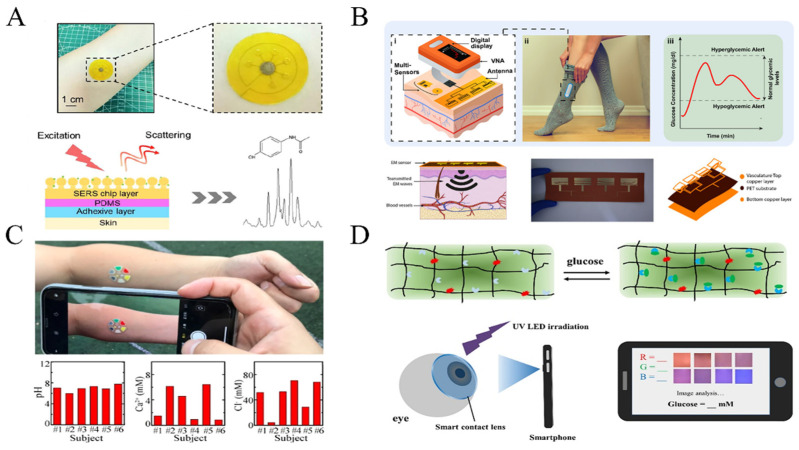
Schematic diagram of non-contact sensing technology and optical sensing technology: (**A**) Wearable SERS sensing technology (adapted from [113]); (**B**) Wearable electromagnetic wave sensing technology (adapted from [112]); (**C**) Colorimetric sensing technology and data analysis (adapted from [114]); (**D**) Fluorescent sensing technology and data analysis (adapted from [115]).

Surface-enhanced Raman spectroscopy (SERS) is a molecular vibration spectrum that utilizes Raman scattering signals for compound molecule detection, and the concentration of target molecules can be analyzed through chemical fingerprints (Figure 5A). The Raman scattering signal is relatively low in intensity, only 10^−10^ of incident light. Therefore, researchers have endeavored to enhance the Raman signal by electromagnetic or chemical enhancement, with a maximum gain of 10^14^, greatly improving detection resolution and accuracy [101,102,103]. Electromagnetic enhancement is mainly contributed by surface plasmon resonance in metal nanostructures, which is usually found in materials such as gold, silver and copper, and the nanoparticle diameter and spacing also affect the enhancement effect. Chemical enhancement relies on the charge transfer between the target molecule and the photon, which enhancement amplitude is much lower than electromagnetic enhancement [104,105].

Wearable detection using SERS has been reported, such as those Zhao et al. developed: core-shell structured Au nanorods (AuNRs@Au) as SERS tags [106]. These SERS tags were deposited in textiles to be used as a kind of wearable sensor. When the textile came into contact with the skin, the lactic acid and glucose molecules in sweat were collected by the SERS tags. The SERS signal to be measured and the standard SERS signal were fitted to a numerical value to obtain the functional equation, and the concentration of glucose and lactate contained in human sweat can be accurately calculated. Despite the promising potential of SERS in molecular concentration analysis, its susceptibility to interference poses a significant challenge in accurately identifying target molecular signals in complex biological fluids. As evidenced by the limited number of publications on using SERS for wearable detection, this technology is still in its nascent stage [107].

#### 3.1.2. Electromagnetic Sensing Technology

The electromagnetic sensing technology measure target molecules concentration based on the dielectric permittivity and its specificity with target concentration (Figure 5B). Resonant and non-resonant methods are commonly employed by researchers to measure dielectric permittivity. Non-resonant methods rely on the transmission characteristics of electromagnetic waves to estimate changes in dielectric permittivity, while resonant methods employ the interaction strength between the electromagnetic wave and the target molecule to characterize dielectric permittivity. Among the broad range of electromagnetic waves, terahertz waves (THz), which range from 0.1 to 10 THz, offer a unique fingerprint spectrum, high transmission, and low energy, and have been utilized for non-invasive detection of metabolites [108].

Baghelani et al. performed non-invasive analysis of lactate concentrations in interstitial fluid based on electromagnetic waves. The chip-less tag resonator and the reader communicate via electromagnetic coupling. The resonant frequency of the chip-less tag fluctuates in proportion to the concentration of lactate in the interstitial fluid, facilitating accurate measurement of lactate concentrations ranging from 1 to 10 mM and evaluation of the aerobic exercise [109]. In addition to lactate, electromagnetic wave sensing is widely applied in glucose monitoring [110,111,112]. However, continuous monitoring of related drug molecules has yet to be reported.

### 3.2. Epidermal Sensing Technology

Bodily fluids, such as sweat, tears, saliva, urine, and exhaled breath condensate, offer valuable insights into health monitoring due to their ability to carry various compounds and establish linear correlations with blood levels, as demonstrated by numerous researchers [116]. Epidermal sensing technologies provide direct detection of target molecules by integrating sensitive components onto the skin or mucosal surface [117]. Sweat, tears, saliva, urine, and exhaled breath condensate are the main biofluids used for detection, as they contain abundant biomarker information. Electrochemical and optical sensing are the most commonly used epidermal sensing technologies due to their high sensitivity and accuracy. Additionally, researchers often employ sensors in the form of skin patches, skin tattoos, wristbands, gloves, glasses, and clothing. These wearable devices not only enable timely contact with secreted bodily fluids but also eliminate the need for additional wearing steps, making them more convenient to use.

#### 3.2.1. The Source of Biofluids

Sweat is primarily produced by sweat glands located in various areas of the body surface, including eccrine, apocrine, and apocrine glands [118]. Sweat secretion is regulated by the central nervous system to maintain thermal homeostasis [119]. An average healthy adult produces around 500–700 mL of sweat per day, making sweat the most accessible biofluid [99]. Sweat is predominantly composed of water (99%), with the remaining 1% containing metabolic waste, micronutrients, and toxicants, specifically including sodium chloride, urea, uric acid, lactic acid, glucose, protein, lipids, cortisol, and more [120,121]. However, obtaining sufficient amounts of sweat can be challenging for patients with chronic diseases, nerve damage, skin disorders, and congenital dysplasia of sweat glands. Researchers have employed several methods to stimulate sweat secretion, including exercise, heat, stress, and ionic stimulation. For instance, Basu et al. used ionophoresis to introduce the agonist pilocarpine, which activates muscarinic receptors to induce sweat production [122]. However, the sweat rates from pilocarpine are transient, so Simmers et al. used the ionotropic agent carbachol, which could maintain sweat rates for over 24 h at a lower dose (<5.25–42 mC cm^−2^) [123]. Other drugs, such as acetylcholine [124] and catecholamines [125], have similar effects. Nonetheless, the ionic composition of the sweat stimulated by these drugs may differ from naturally secreted sweat [126]. To address the challenge of insufficient sweat secretion in patients, it is essential to develop alternative biofluids that contain similar metabolites and drugs for detection.

Tears are a hypotonic ultrafiltrate of blood and thus have a high correlation with biomarker concentrations [127,128]. Compared to sweat, tears are notably rich in proteins, including lactoferrin, lysozyme, albumin, and thousands of other proteins. Alongside these, tears contain various metabolites such as ascorbic acid, glucose, cholesterol, and uric acid, which can provide researchers with personalized data on health monitoring [129]. Unlike sweat, tears are continuously secreted into the eye fundus at approximately 1.2 μL per minute making them a convenient and less environmentally influenced option for biomarker analysis [130]. Although current tear-based biomarker analysis primarily focuses on glucose concentration, the potential for monitoring other biomarkers and drug molecules is significant and promising.

Saliva is a clear, colorless liquid that is secreted by the salivary glands located in the oral cavity. This biological fluid is a rich source of biomarkers, including enzymes, vitamins, urea, uric acid, free amino acids, and drugs, which can be detected after their administration [131,132]. Saliva is produced in substantial quantities, up to 1.5 L per day, and is highly correlated with biomarker blood concentrations [133]. One of the most significant advantages of saliva collection is its simplicity, painlessness, and safety. Due to the mouth’s anatomical structure, saliva readily contacts the sensing element, eliminating the need for microfluidic channels in sampling collection. Nevertheless, it should be noted that many drugs are ingested orally, and saliva as a testing sample may be contaminated, leading to measurement errors. As such, this factor must be taken into account during the testing process.

Exhaled breath condensate (EBC) has emerged as a non-invasive substrate for biomarker detection since the 1970s. Some biomarkers are excreted not only in solution but also in the form of aerosols through normal breathing [134,135]. EBC is collected by condensing respiratory aerosols and contains abundant salts, lipids, protein biomarkers, virus and pathogen particles, and a biological matrix of over 250 volatile biomarkers. It offers the possibility of continuous and non-invasively monitoring the drug related biomarkers [136,137]. However, the concentration of drug molecules in exhaled breath is often 1000 to 10,000 times lower than in blood samples, making the use of highly sensitive detection devices necessary [138,139]. Despite this limitation, EBC holds great promise as a non-invasive and potentially informative biofluid for biomarker detection.

##### Correlation of Compound Concentration between Biofluids and Blood

Sampling biofluids is a non-invasive procedure that does not cause wounds. However, the biomarker concentration correlation between these fluids and blood is a crucial factor that must be considered. For instance, methylphenidate is a stimulant used for treating ADHD in children. Overdose of methylphenidate can cause various adverse effects, including mental anxiety, behavioral disturbances, and visual disturbances. In order to address the lack of appropriate biological matrices for drug therapy monitoring, researchers investigated the correlation of methylphenidate concentration between saliva and blood. They found a concentration ratio of one-tenth and a consistent range of changes maintained within four weeks, demonstrating the feasibility of saliva testing of methylphenidate [140]. Vasudev et al. also established the correlation between carbamazepine levels in blood and saliva using high-performance liquid chromatography, with a correlation coefficient of 0.659 (*p* < 0.001) [141]. Similar correlations were observed for oxazepam [142], leflunomide [143], dolutegravir [144], and valproic acid [145]. However, the blood–biofluids correlation investigated drugs are limited in few types, and researchers continue to expand drug monitoring and lay the groundwork for subsequent contact wearable sensing.

#### 3.2.2. Optical Sensing Technology

Wearable optical sensors are based on color change mechanisms between chromophores or fluorophores and target molecules in biofluids, allowing direct quantitative analysis through image analysis software [146]. Current colorimetric sensing methods primarily rely on the redox reactions between enzymes and their substrates (Figure 5C). For example, glucose can be measured through the cascade catalysis by enzymes resulting in o-dianisidine turning blue and iodine turning brown [147]. In the presence of cofactor NAD^+^ (nicotinamide adenine dinucleotide), lactate can be catalyzed by lactate dehydrogenase to generate electrons, yielding formazan dyes of yellow color [148]. The creatinine enzyme and peroxidase-catalyzed creatinine acid convert 4-aminophenanthrene-4-aminophenazone to a purple-red complex [149]. The resulting color changes of chromogenic agents are often translated into RGB values to quantify the intensity of the color. However, it is important to note that RGB values may be susceptible to ambient light when capturing images, causing measurement deviations.

The development of colorimetric sensing technology is hindered by the poor stability and high cost of native enzymes used in the sensor. To address this issue, some researchers have explored the use of nanomaterials with natural enzyme-like properties. For example, Li et al. immobilized Prussian blue nanoparticles on Au nanowires to develop a tape for detecting uric acid in sweat [150]. While such nanoparticles offer improved stability, they do not alter the colorimetric sensing mechanism. Additionally, the limited availability of commercial chromogenic reagents further restricts the detection range of colorimetric sensors. Therefore, the development of colorimetric sensors for continuous TDM requires further exploration and development.

Wearable fluorescence sensing is considered more sensitive and has lower detection limits than colorimetric sensing (Figure 5D). Unlike most colorimetric sensing methods that rely on enzymatic reaction catalysis, fluorescent sensors can be covalently bound to biomarkers without intermediaries, making them suitable for detecting various molecules and ions in tears and sweat. For instance, Deng et al. developed boronic acid-modified anthracene derivatives that specifically bind glucose and emit blue fluorescence, achieving a detection limit of up to 85 μM [115]. Badugu et al. prepared wearable fluorescent sensors by depositing water-soluble fluorescent probes to silicon hydrogel (SiHG) contact lenses, successfully measuring six ions in tears, including pH (H_3_O^+^/OH^−^), Na^+^, K^+^, Ca^2+^, Mg^2+^, and Cl^−^ to investigate the pathogenesis of dry eye disease [151]. Additionally, lanthanides, which have a high quantum yield and long emission lifetime, can also serve as fluorescent probes. Xu et al. used them to detect Cl^−^ [152]. However, fluorescent probes are susceptible to interference from their own fluorescence emission, leading researchers to explore up-conversion technology. Hu et al. embedded up-conversion nanoparticles into polyacrylamide hydrogel to fabricate wearable fluorescent sensors for detecting sweat urea, achieving highly sensitive fluorescence detection due to its high tissue transmittance [153].

Compared with colorimetric sensing technology, although fluorescent sensing technology has more advantages in sensitivity, its disadvantages are also obvious. For example, most fluorescent materials need to be measured in a dark environment to obtain more accurate results. If they are exposed to strong light for a long time, photobleaching is likely to occur. Fluorescence analysis often requires complex light sources, which brings some inconvenience to the measurement.

#### 3.2.3. Electrochemical Sensing Technology

Among the various wearable sensing technologies currently developed, electrochemical technology stands out due to its numerous advantages, including high sensitivity, rapid reaction speed, and excellent linearity [154,155,156]. Consequently, it has become the most commonly employed sensing technology in many applications. In electrochemical sensing technology for wearable monitoring, researchers typically immobilize a bio-sensitive element onto a transducer and analyze the resulting signal change in current or potential to determine the concentration variation of the target molecule. These bio-sensitive elements often comprise enzymes, aptamers, antibodies, and other similar entities. The utilization of electrochemical technology in wearable sensing systems holds great promise for diverse applications, providing reliable and accurate monitoring capabilities while maintaining compatibility with the skin.

##### Conventional Electrodes without Recognition Element Sensing Technology

Conventional electrodes without recognition elements directly quantify the electrochemical activity of target molecules without relying on recognition elements. They often improve detection sensitivity by doping electrocatalysts with rich redox sites. Sun et al. deposited nitrogen-doped NiCoO_2_ nanosheets on a carbon-fiber substrate to prepare a wearable enzyme-free sensor, which exhibits excellent electrocatalytic properties and high stability towards glucose with a sensitivity of 592 μA mM^−1^ [157]. Similarly, Wang et al. immobilized boron-doped graphene quantum on carbon nanotubes as additional redox reaction sites, achieving a sensitivity of up to 8.92 μA μM^−1^ cm^−2^ for uric acid detection [158]. Notably, previous studies have mainly focused on endogenous metabolites in the body, but some researchers are now exploring the possibility of continuous drug monitoring using electrochemical electrodes. Several electrochemically active drugs, such as sulphadiazine [159], sulfasalazine [160], and ornidazole [161], can be directly detected by electrodes, allowing for the determination of their concentrations. These pioneering studies have set the stage for the future development of wearable sensors for continuous drug monitoring.

##### Ion-Selective Electrodes Sensing Technology

**Figure 6:**
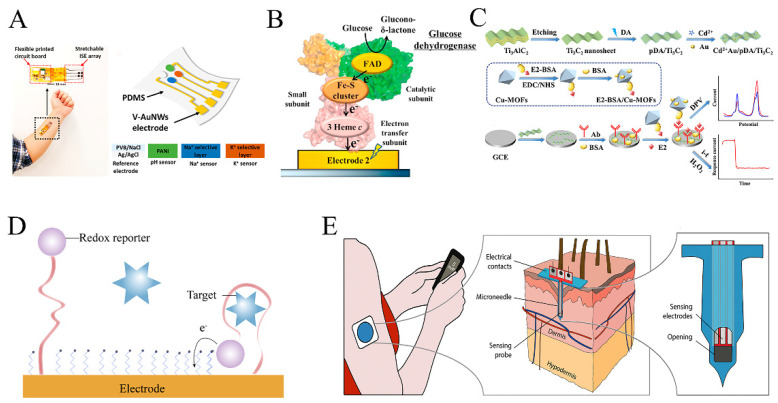
Schematic illustration of the electrochemical biosensing technology: (**A**) Electrochemical sensing technology based on ion selective electrodes (adapted from [180]); (**B**) Enzyme based electrochemical sensing technology (adapted from [172]); (**C**) The dual-mode electrochemical competitive immunosensing technology(adapted from [177]); (**D**) Electrochemical aptamer-based sensing technology; (**E**) Invasive electrochemical sensing technology using interstitial fluid as detection sample (adapted from [181]).

Ion-selective electrodes rely on the membrane potential to selectively determine the activity or concentration of ions in solution [162] (Figure 6A). The potential difference generated by the selective transport of ions through the membrane can be quantitatively measured using the Nernst equation. Various ion-exchange carriers, such as conductive polymers, carbon-based materials, and nanomaterials, have been employed to prepare ion-selective electrodes [163]. Current research on ion-selective electrodes has mainly focused on health monitoring parameters, such as Na^+^, K^+^, and Pb^2+^ [99]. For instance, Lim et al. employed ion carriers including sodium tetrakis3,5-bis(trifluoromethyl)phenyl borate (NaTFPB) and sodium tetrakis3,5-bis(trifluoromethyl)phenylborate (KTClPB) to prepare sodium ion-selective electrodes with a detection range of 6.5–11.8 mM [164]. In drug monitoring, only lithium ions for bipolar affective treatment have been successfully developed. Platinum nanostructures were deposited on a PI film, and a lithium-selective membrane was dropwise added to the working electrode, enabling the detection of lithium ions in artificial sweat with a sensitivity reaching 56.8 mV decade^−1^ [165]. Although ion-selective electrodes hold great potential for continuous drug monitoring, developing novel ion exchange carriers remains crucial for further advancement in drug administration.

##### Enzyme-Based Electrochemical Sensing Technology

Enzyme-based electrochemical sensing is a widely used method for detecting target molecules by modifying enzymes on working electrodes to specifically recognize target molecules and generate corresponding electrical signals (Figure 6B). The sensitivity of enzyme-based sensing depends on the stability of the enzyme, the immobilization efficiency, and the electron transfer rate to the working electrode [166]. Enzyme-based electrochemical amperometric and potentiostat sensors have been developed to measure many metabolites [167], and glucose oxidase (GOx) electrochemical sensing technology is currently the most developed and commercialized [168,169]. In addition, lactate oxidase, uricase, and β-hydroxybutyrate dehydrogenase have been developed for lactate, uric acid, and ketone monitoring. For continuous drug monitoring, β-lactamase enzymes and tyrosinase enzymes have been developed to monitor β-lactam antibiotics and L-Dopa, respectively. Moreover, organophosphorus hydrolase (OPH) enzymes have been used in organophosphorus pesticide assays to prevent irreversible damage caused by exposure to high concentrations of organophosphorus pesticides.

Enzyme-based sensing technologies are highly promising for the detection of metabolites, and the development of stable and efficient enzyme immobilization methods is a key focus of current research [170]. One example of successful immobilization is the creation of highly stable single-molecule enzyme nano-capsules by Dhanjai et al. [171]. In this method, GOx was encapsulated within a thin, porous polymerizable vinyl/acryloyl shell, retaining 86% of its native enzyme activity. In addition, a wearable multiplexed biosensor has been designed by Hiraka et al. which incorporates a fusion enzyme containing l-lactate oxidases and b-type cytochrome proteins [172]. This fusion enzyme is immobilized on a gold electrode, and direct electron transfer between the l-lactate oxidases and b-type cytochrome proteins is used to measure lactate and glucose in sweat simultaneously. The glucose and lactate sensing ranges were found to be 0.1–5 mM and 0.5–20 mM, respectively [173,174], providing more accurate and scientific drug use guidance for diabetic patients.

##### Electrochemical Immunosensing Technology

Most antigens and antibodies are proteins that exhibit structural complementarity, forming antigen-antibody complexes binding. Electrochemical immunosensing technology, based on the specific interactions between antigens and antibodies, utilizes electrochemical detection methods to analyze biomarkers and drugs in biofluids (Figure 6C). According to the signal conversion scheme, they could be divided into two types: direct type and indirect type. In the direct type, immune signals are directly converted into electrical signals during antigen-antibody recognition. On the other hand, the indirect type involves converting the combined antigen-antibody information into intermediate information, which is then further converted into electrical signals. Existing electrochemical immunosensors are limited to single-point measurements of target molecules in vitro. Among the molecules studied extensively in this technology, cortisol and other metabolites have received significant attention [175,176,177]. These studies lay the groundwork for future wearable sensors capable of continuously monitoring drug molecules.

Anti-cortisol antibodies are immobilized on SnO_2_ nanoflake-integrated conductive carbon fiber (SnO_2_/CCY) via non-covalent bonding interactions, providing more binding sites for antibodies. This configuration offers a linear range of detection from 10 fg mL^−1^ to 1 µg mL^−1^ [178]. Recently, a disposable electrochemical immunesensor was used to detect 25-hydroxyvitamin D3 (25(OH)D3), the metabolite of vitamin D, in saliva and serum. The sensor employed tree-like gold dendrite nanostructures (AuDdrites) with a high specific surface area and active sites. L-cysteine (L-cys) was adsorbed onto AuDdrites to provide selective binding sites, while anti-25(OH)D3 antibodies served as the sensing units, binding to L-cys. K_4_[Fe(CN)_6_] was utilized as an oxidative fluorescent probe to quantify the concentration of 25(OH)D3, exhibiting a linear range from 0.1 to 900 ng mL^−1^ [179]. These studies will inspire researchers to develop wearable electrochemical immunosensors with expanded detection ranges, providing potential monitoring methods for continuous drug monitoring in vivo.

##### Electrochemical Aptamer-Based (E-AB) Sensing Technology

Aptamers are single-stranded nucleic acids with programmable structures, high affinity, and the ability to bind reversibly to various molecules [30]. E-AB sensing technology involves the immobilization of aptamers onto the surface of an electrode. Upon binding to the target molecule, the electron transfer rate or interfacial properties are altered, leading to a measurable electrochemical signal (Figure 6D). E-AB sensors have become a popular tool in drug analysis and biosensing due to their sensitivity and selectivity. While the E-AB sensor has been successful in many assays, it is limited by a low response signal due to the low number of electrons provided by the redox reporter and the limited contact area of the sensing electrode. To address this, shrinkage-induced wrinkled gold films have been used to increase the surface area of sensing electrodes, leading to a ten-fold increase in the current signal and a two-order of magnitude improvement in the detection limit compared to smooth gold films [182]. Additionally, nanoparticles [183] and multi-walled carbon nanotubes [184] have been employed to increase the microscopic surface area of electrodes. Researchers have also developed stronger covalent bonds to enhance the stability of the signal output, including modifying carbon surfaces through grafting primary aliphatic amine modification strategies, which form carbon-nitrogen bonds and increase the bond strength by up to four times [185]. They modified carbon surfaces by grafting primary aliphatic amine modification strategies, forming carbon-nitrogen bonds and increasing the bond strength up to four times. Moreover, researchers have attempted to improve the signal output of electrochemical aptamers by developing new redox probes [186], enhancing the ionic strength of buffers, and using organic electrochemical transistors [187,188]. Several aptamers for drugs have also been developed and tested in vitro, such as insulin aptamer [189], vancomycin aptamer [190], and digoxin aptamer [191].

Compared to enzyme-based electrochemical sensors and electrochemical immunosensors, E-AB sensors remain highly versatile and capable of screening a broad range of markers and drugs, making them a promising technology for wearable drug monitoring. To realize this potential, several issues must be overcome, including improving signal sensitivity, optimizing aptamer screening, developing novel redox probes, and achieving miniaturization. By addressing these challenges, E-AB sensors have the potential to revolutionize the field of wearable drug monitoring, offering an accessible, convenient, and accurate method for monitoring a wide range of biomarkers and therapeutic agents in real-time.

### 3.3. Invasive Sensing Technology

Invasive sensing technology offers direct access to the body’s tissues, enabling contact with blood or interstitial fluid, containing a wide range of compounds. By monitoring these biofluids, concentration changes of target molecules can be detected in real-time. The depth of penetration varies depending on the type of test sample. When using interstitial fluid as the test sample, the invasion depth is typically greater than 2.3 μm, which corresponds to the thickness of the stratum corneum [192]. In the case of sampling blood, the sensor must be implanted into a superficial vein, usually in the upper limb, requiring a deeper level of invasion. Wearable invasive sensing technology was initially employed for blood glucose detection and has since evolved to monitor drug metabolism within the body. It can also be integrated with drug delivery systems, presenting novel opportunities for precision medicine [193].

#### 3.3.1. The Source of Biofluids

Blood is a vital component in the circulatory system, responsible for transporting various nutrients and metabolites to maintain normal physiological function. When administering drugs, regardless of the administration method, they must be distributed through the bloodstream to reach different tissues and organs, exert their therapeutic effects, and eventually be excreted from the body. Therefore, blood is considered the most reliable and convenient sample source for monitoring therapeutic drugs. In comparison to other biofluids, using blood as a wearable detection sample offers the advantage of minimal physiological time delay, enabling timely reflection of changes in drug concentration within the body.

Interstitial fluid, as a crucial extracellular fluid surrounding cells, plays a significant role in material exchange between blood and cells [194]. It serves as a valuable resource for analyzing metabolites and drugs, facilitating the transfer of signaling molecules, antigens, and cytokines between different compartments [195]. The continuous flow of interstitial fluid without clotting, along with its real-time tracking capabilities for target molecule concentrations, make it well-suited for personalized assessment and monitoring of the body’s physiological functions. The collection of interstitial fluid is primarily achieved through the use of microneedles, leveraging capillary forces between the fluid flow and microneedles to obtain real-time information regarding marker molecules [196,197].

#### 3.3.2. Optical and Electrochemical Sensing Technology

Both invasive sensing technology and contact sensing technology operate on the same principle, utilizing electrochemical or optical technology to gather information about target molecules. In the case of interstitial fluid as the detection sample and electrochemical sensing technology as the detection method, microneedles serve as solid supports with modifications of sensitive elements such as enzymes, antibodies, or aptamers at their tips (Figure 6E). Typically, microneedles have dimensions of approximately 100−600 μm in length and 50–200 μm in width, allowing them to penetrate the stratum corneum and epidermis and reach the dermis to access the interstitial fluid. Since microneedles avoid contact with nerves in the dermis, they minimize skin irritation and inflammation and promote rapid wound healing [198]. Microneedles can be fabricated through various methods, including 3D printing, soft lithography, and template modeling, resulting in single or arrayed micro-structures [199]. Substrate materials for microneedles commonly involve metals, silicon [200,201], and conducting polymers, which possess high electrical conductivity and are amenable to biological material modifications.

Prior to their use in target molecular assays, microneedles need to be functionalized with sensitive elements. For example, the immobilization of the β-Lactam enzyme onto the microneedles enables the detection of penicillin in interstitial fluids. In this biosensor, a pH-sensitive iridium oxide layer is coated on the surface, allowing the detection of pH changes resulting from the β-Lactam enzymatic hydrolysis of penicillin [57]. In optical sensing technology, sensitive elements are implanted in the body to enable continuous drug monitoring. In the case of doxorubicin, a chemotherapy drug known for its strong affinity for DNA and its ability to disrupt cell proliferation, single-walled carbon nanotubes functionalized with DNA were utilized to measure changes in the concentration of doxorubicin in interstitial fluid. The measurement of doxorubicin concentration at specific body tissues was achieved through the red-shifting of the emission and excitation wavelengths of carbon nanotube photoluminescence [202].

In the case of using blood as a test sample, flexible electrodes are delivered to the blood vessels through catheters with an outer diameter typically less than 0.6 mm [203]. These electrodes come into contact with drug molecules in the blood. However, this technology requires the implantation of sensors in the vein, which can induce inflammatory reactions. To mitigate this issue, researchers have incorporated anti-inflammatory materials and performed chemical surface modifications on the electrode surface to reduce inflammatory responses. Furthermore, flexible materials have been employed to minimize the mismatch between the rigid implantable surface and the soft living tissue [204]. Aside from the aforementioned challenges, the complexity of blood composition, including sugars, lipids, and proteins, can introduce interference during detection. Only a limited number of articles have reported continuous detection of drug concentrations in blood [30,59], indicating that sensing technology based on blood as a monitoring sample requires further development.

Overall, wearable electrochemical sensing technologies have been extensively studied for non-invasive and invasive monitoring of drug concentration at the molecular level. However, despite its advancements, electrochemical sensing technology still possesses several limitations. The predominant detection methods, potentiometry and chronoamperometry, often result in inadequate stability and repeatability of measurements. Therefore, further advancements in electrochemical biosensor construction are necessary to diversify detection methods, enhance detection sensitivity and selectivity, and progress towards automation, miniaturization, and multifunctionality.

### 3.4. Emerging Sensing Technologies and Application Barriers

With the aging population, the rise of new diseases, and the spread of epidemics, there has been a significant increase in drug usage. Wearable sensing technologies have been employed in patient monitoring to minimize side-effect toxicity and maximize therapeutic effectiveness. In hospitals, wearable sensors worn by acute critical patients can enhance the safety and efficiency of emergency rooms, alleviating the pressure on medical staff [205]. Long-term treatment data collected from patients can aid doctors in assessing recovery and optimizing medical resource allocation. At home, wearable sensors enable the convenient and non-invasive capture of real-time drug concentration in the body, eliminating the need for tedious hospital testing procedures and reducing patient discomfort. Additionally, long-term data uploaded to the cloud allow remote assistance from doctors for timely medication dosage optimization and treatment evaluation.

Despite the advancements in wearable sensor technologies, there are still practical challenges to overcome. Achieving a balance between accuracy and non-invasiveness remains difficult, requiring careful consideration for each technology’s application. Non-contact sensing technology offers the advantage of minimizing sensor contamination by biofluids, as it does not require direct contact with the body. However, it faces limitations in capturing weak output signals due to external interferences such as environmental noise, motion artifacts, and optical impedance [17]. Traditional colorimetric and fluorescence sensing technologies, known for their simplicity and battery free capability, mainly rely on enzyme catalysis or direct redox reactions to monitor a limited number of target molecules through simple analyses. Nonetheless, these approaches encounter significant challenges in terms of stability and reversibility, posing obstacles for successful implementation in wearable drug monitoring. Electrochemical sensing methods, the most mature among the options, offer high detection sensitivity and fast reaction speeds. However, practical measurements must account for factors such as electrical impedance, electrical noise on the skin surface, and the lifespan of sensitive components. Researchers have explored strategies to provide a stable working environment for electrodes, including the use of flexible electrodes and conductive hydrogels to enhance electrode-skin adhesion [206,207]. Modifications with functional materials have been investigated to improve anti-interference capabilities, and data algorithms have been developed to promptly correct electrode signals.

Therefore, continuous optimization of existing sensing technologies is essential, focusing on improving both the accuracy and non-invasiveness in monitoring target molecules. Regarding detection accuracy, the ultimate objective of drug monitoring is to achieve optimal therapeutic effects by identifying pharmacokinetic biomarkers. In addition to directly monitoring drug molecule concentrations, exploring stable metabolite molecules that indirectly reflect changes in drug concentration can enhance detection accuracy. Utilizing artificial intelligence technology to analyze drug pharmacokinetics and gather data from a large number of test samples can address individual variations. Moreover, leveraging high-precision industrial-scale manufacturing technology can multiply sensing channels, enabling simultaneous multi-modal monitoring of target molecules and reducing detection errors. Furthermore, non-invasive wearable sensing technology continues to advance, utilizing biofluids such as sweat, tears, and saliva as test samples to analyze drug molecules for non-invasive and continuous pharmacokinetic monitoring. Non-invasive monitoring eliminates the invasive wounds and complications, reduces patient discomfort, and improves patient satisfaction. Overall, wearable sensing technology is a thriving field in the medical industry, facilitating real-time monitoring of patients’ pharmacokinetics through intelligent devices. This advancement holds the potential for groundbreaking discoveries in medicine, pharmaceuticals, and vaccine development.

## 4. Discussion

Challenge: This review offers a comprehensive overview of the advancements in wearable sensors for drug monitoring, encompassing biofluids, sensing technologies, and types of drugs suitable for wearable monitoring. Despite the immense potential of wearable sensors in drug monitoring, several significant challenges persist that need to be addressed in order to establish reliable and effective monitoring systems. Key challenges include establishing correlations between drug concentrations in peripheral biofluids and blood, as well as developing novel sensing materials and technologies. Although some drugs have successfully correlated with peripheral biofluid concentrations, further investigations are required, encompassing long-term and comparative measurements conducted under diverse environmental conditions. Ensuring the safety and efficacy of therapeutic drug monitoring necessitates wearable sensors that deliver highly accurate and reliable results while maintaining stability, specificity, and flexibility over extended periods of use.

Prospects: Wearable sensors offer enormous possibilities for personalized medicine by enabling continuous monitoring of drug content in biofluids and dynamic adjustment of drug intake to achieve more effective therapeutic outcomes. Existing research has demonstrated the immense potential of wearable sensors in drug monitoring. In the foreseeable future, highly integrated multimodal wearable sensors can be utilized to simultaneously monitor multiple drugs, expanding the scope of drug monitoring and enhancing detection accuracy. Furthermore, these sensors can establish feedback pathways for drug detection and delivery, facilitating integrated diagnosis and treatment approaches. In the era of digital medicine, wearable sensors supported by the Internet of Things (IoT) can transmit real-time monitoring data to cloud servers, thereby eliminating the risk of data loss. This capability also proves invaluable in areas with limited medical resources, as it enables remote diagnosis for patients and timely adjustments to medication plans. Overall, wearable sensors hold the potential to innovate existing medical methods and revolutionize the field of medicine in the era of digital healthcare.

## Figures and Tables

**Table 1:** The concentration of drugs that require treatment monitoring in biofluids.

Type of Drugs	Compound	Martrix	Biofluid Level	Ref
Immunosuppressants	Tacrolimus	Serum	0.01–0.015 μg mL^−1^	[20]
	Cyclosporin	Serum	80–1000 μg mL^−1^	[21,22]
Antiepileptic	Carbamazepine	Serum	6000–8000 μg mL^−1^	[23]
	Phenytoin sodium	Serum	10–20 μg mL^−1^	[24,25]
	Phenobarbital	Serum	10–40 μg mL^−1^	[26]
	Valproic acid	Serum	50–100 μg mL^−1^	[27]
	Lamotrigine	Serum	2.5–15 μg mL^−1^	[28]
	Levetiracetam	Serum	12–46 μg mL^−1^	[28]
Antimicrobial drugs	Vancomycin	Serum	0.005–0.04 μg mL^−1^	[29]
		Sweat	8.7–50.7 μg mL^−1^	[30]
	Meropenem	Serum	8–32 μg mL^−1^	[31]
	Linezolid	Serum	2–7 μg mL^−1^	[32]
		ISF	0.101–1.2 μg mL^−1^	[33]
	Tobramycin	Serum	4–6 μg mL^−1^	[34]
	Voricnazole	Serum	0.5–5 μg mL^−1^	[32]
Cardioactive drugs	Digoxin	Serum	0.001–0.0025 μg mL^−1^	[35]
Antidepressants	Lithium	Serum	44.4–66.6 μg mL^−1^	[36]
Analgesics drugs	Fentanyl	Serum	1–3 μg mL^−1^	[37]
		Sweat	0.17–1.02 μg mL^−1^	[38]
	Methadone	Serum	0.08–0.7 μg mL^−1^	[39]
		Sweat	120–2160 ng patch^−1^	[40]
Anti-asthmatic drugs	Theophylline	Serum	5–15 μg mL^−1^	[41]
Antipsychotic drugs	Clozapine	Serum	0.35–0.5 μg mL^−1^	[42]
	Risperidone	Serum	0.02–0.06 μg mL^−1^	[42]
	Perphenazine	Plasma	0.0012–0.0024 μg mL^−1^	[43]
	Fluphenazine	Plasma	0.0002–0.002 μg mL^−1^	[43]
	Thiothixene	Plasma	0.002–0.015 μg mL^−1^	[43]
	Olanzapine	Serum	0.002–0.004 μg mL^−1^	[42]

## Data Availability

Data are contained within the article.
